# Medulloblastoma therapy generates risk of a poorly-prognostic H3 wild-type subgroup of diffuse intrinsic pontine glioma: a report from the International DIPG Registry

**DOI:** 10.1186/s40478-018-0570-9

**Published:** 2018-07-26

**Authors:** Hunter C. Gits, Maia Anderson, Stefanie Stallard, Drew Pratt, Becky Zon, Christopher Howell, Chandan Kumar-Sinha, Pankaj Vats, Katayoon Kasaian, Daniel Polan, Martha Matuszak, Daniel E. Spratt, Marcia Leonard, Tingting Qin, Lili Zhao, James Leach, Brooklyn Chaney, Nancy Yanez Escorza, Jacob Hendershot, Blaise Jones, Christine Fuller, Sarah Leary, Ute Bartels, Eric Bouffet, Torunn I. Yock, Patricia Robertson, Rajen Mody, Sriram Venneti, Arul M. Chinnaiyan, Maryam Fouladi, Nicholas G. Gottardo, Carl Koschmann

**Affiliations:** 1 Department of Pathology, Michigan Medicine, Ann Arbor, MI 48109 USA; 2 Department of Pediatrics, Division of Pediatric Hematology-Oncology; Michigan Medicine, Ann Arbor, MI 48109 USA; 3 Department of Haematology and Oncology, Princess Margaret Hospital for Children, Perth, WA 6840 Australia; 4 Michigan Center for Translational Pathology, Michigan Medicine, Ann Arbor, MI 48109 USA; 5 Ontario Institute for Cancer Research, MG5 0A3, Toronto, ON Canada; 6 Department of Radiation Oncology; Michigan Medicine, Ann Arbor, MI 48109 USA; 7 Department of Computational Medicine and Bioinformatics; Michigan Medicine, Ann Arbor, MI 48109 USA; 8 Department of Biostatistics, University of Michigan School of Public Health, Ann Arbor, MI 48109 USA; 9 Division of Radiology, Cincinnati Children’s Hospital, Cincinnati, OH 45229 USA; 10 Division of Oncology, Cincinnati Children’s Hospital, Cincinnati, OH 45229 USA; 11 Division of Biomedical Informatics, Cincinnati Children’s Hospital, Cincinnati, OH 45229 USA; 12 Division of Pathology, Cincinnati Children’s Hospital, Cincinnati, OH 45229 USA; 13 Department of Pediatrics, Division of Oncology, Seattle Children’s Hospital, Seattle, WA 98105 USA; 14 Department of Pediatrics, Division of Haematology/Oncology, Hospital for Sick Children, Toronto, ON M5G 1X8 Canada; 15 Department of Radiation Oncology, Massachusetts General Hospital, Boston, MA 02114 USA; 16 Department of Pediatrics, Division of Neurology; Michigan Medicine, Ann Arbor, MI 48109 USA; 17 Department of Pediatrics, Division of Oncology, Cincinnati Children’s Hospital, Cincinnati, OH 45229 USA; 18 Kids Cancer Centre, Telethon Kids Institute, Subiaco, WA 6008 Australia; 19 Division of Paediatrics, University of Western Australia, Crawley, WA 6009 Australia

**Keywords:** Secondary malignant neoplasm, Diffuse intrinsic pontine glioma, Medulloblastoma, Cranial irradiation, Brainstem

## Abstract

With improved survivorship in medulloblastoma, there has been an increasing incidence of late complications. To date, no studies have specifically addressed the risk of radiation-associated diffuse intrinsic pontine glioma (DIPG) in medulloblastoma survivors. Query of the International DIPG Registry identified six cases of DIPG with a history of medulloblastoma treated with radiotherapy. All patients underwent central radiologic review that confirmed a diagnosis of DIPG. Six additional cases were identified in reports from recent cooperative group medulloblastoma trials (total *n* = 12; ages 7 to 21 years). From these cases, molecular subgrouping of primary medulloblastomas with available tissue (*n* = 5) revealed only non-WNT, non-SHH subgroups (group 3 or 4). The estimated cumulative incidence of DIPG after post-treatment medulloblastoma ranged from 0.3–3.9%. Posterior fossa radiation exposure (including brainstem) was greater than 53.0 Gy in all cases with available details. Tumor/germline exome sequencing of three radiation-associated DIPGs revealed an H3 wild-type status and mutational signature distinct from primary DIPG with evidence of radiation-induced DNA damage. Mutations identified in the radiation-associated DIPGs had significant molecular overlap with recurrent drivers of adult glioblastoma (e.g. *NRAS*, *EGFR*, and *PTEN*), as opposed to epigenetic dysregulation in H3-driven primary DIPGs. Patients with radiation-associated DIPG had a significantly worse median overall survival (median 8 months; range 4–17 months) compared to patients with primary DIPG. Here, it is demonstrated that DIPG occurs as a not infrequent complication of radiation therapy in survivors of pediatric medulloblastoma and that radiation-associated DIPGs may present as a poorly-prognostic distinct molecular subgroup of H3 wild-type DIPG. Given the abysmal survival of these cases, these findings provide a compelling argument for efforts to reduce exposure of the brainstem in the treatment of medulloblastoma. Additionally, patients with radiation-associated DIPG may benefit from future therapies targeted to the molecular features of adult glioblastoma rather than primary DIPG.

**Electronic supplementary material:**

The online version of this article (10.1186/s40478-018-0570-9) contains supplementary material, which is available to authorized users.

## Introduction

Medulloblastoma is the most common malignant pediatric brain tumor, and standard treatment includes surgical resection followed by adjuvant external beam radiation therapy (EBRT) and systemic chemotherapy [14]. As the prognosis of medulloblastoma has improved, late complications such as secondary malignant neoplasms (SMNs) have increased in frequency [15, 39]. While 10-year survival rates of medulloblastoma are now near 80%, the 20-year cumulative incidence of SMNs is reported to be as high as 20%, comprising 11.8% of late mortality [15, 29, 31, 39].

The elevated risk of SMNs in medulloblastoma survivors may be due to high doses of EBRT. The risks of glioma, the most common SMN reported after primary medulloblastoma, increase linearly with radiation dose [9, 19, 31, 42]. Radiation dosing for medulloblastoma varies based on clinical and molecular risk stratification, and standard treatment involves craniospinal irradiation (CSI) with a posterior fossa boost. No previous studies have assessed the risk of the development of radiation-associated DIPG in medulloblastoma survivors, which could impact the future dose and modality of radiation therapy in future clinical trials.

Diffuse intrinsic pontine glioma (DIPG) is a rare infiltrative brainstem tumor, and patients rarely survive longer than 2 years after diagnosis. DIPG is diagnosed primarily by radiographic features showing an intrinsic lesion that encompasses at least 50% of the pons. When available, histology frequently shows features consistent with an infiltrating high-grade glioma (HGG). Approximately 80% of these tumors harbor a point mutation in the histone H3 (H3.3 and H3.1), which now defines the new histomolecular entity Diffuse Midline Glioma, H3K27M-mutant, and is associated with epigenetic dysregulation of neuro-developmental pathways and a worse prognosis than H3 wild-type DIPG [8]. Recent studies suggest that H3 mutants are distinct biological entities, and that H3.3 mutants alone may display a worse prognosis relative to H3 wild-type [10, 20].

There is a paucity of data specifically addressing the risk and molecular characteristics of radiation-associated DIPG among medulloblastoma survivors. A recent report performed genomic analysis of recurrent tumors of seventeen pediatric medulloblastoma patients [33]. The report revealed some of the tumors as secondary glioblastomas with known driver mutations and identified *PDGFRA* as a potential molecular target for therapy. Although this work addressed radiation-associated cancers following treatment for pediatric medulloblastoma, there were no pontine tumors and there remains no published incidence data for radiation-associated DIPG. Here, this report describes a poorly-prognostic molecular subgroup of H3 wild-type DIPG that occurs as a not infrequent complication of radiation therapy in survivors of pediatric medulloblastoma.

## Materials and methods

### Case acquisition

The International DIPG Registry (IDIPGR) was queried for cases of DIPG diagnosed after radiation treatment for primary medulloblastoma. Details of the registry’s structure and recruitment are described elsewhere [12]. Diagnosis of DIPG was confirmed by central radiology review by two primary neuroradiologists (BJ, JL). A Medline/PubMed and Google Scholar search was performed to identify any additional published cases. Various combinations of keywords were used including: *medulloblastoma, diffuse intrinsic pontine glioma, brainstem glioma, pontine glioma, secondary malignant neoplasm.* Articles dated from 1999 to 2017 were obtained and demographic, treatment, and survival data extracted as available. All patients diagnosed with primary medulloblastoma from age 0–21 years who were subsequently diagnosed with brainstem glioma were included. While it was not possible to review imaging for all cases obtained from primary literature, care was taken to exclude patients with focal (non-diffuse) brainstem tumors.

### Methylation analyses of primary medulloblastoma

The medulloblastoma methylation-derived subgrouping was performed using the Infinium Assay with the Illumina MethylationEPIC BeadChip platform. DNA was extracted and isolated according to standard protocols, and bisulfite conversion was performed using the Zymo EZ DNA methylation kit. A support vector machine was trained on a cohort of medulloblastoma samples to develop a methylation-derived sub-classification prediction algorithm. The MethylationEPIC BeadChip 46 CpG dinucleotide signature algorithm and R statistical program (version 3.0.0) were used to classify the medulloblastoma tumor into one of four subgroups: Sonic hedgehog pathway activated (SHH), Wnt-pathway activated (WNT), Group 3, or Group 4. Quality control parameters were assessed using Illumina Genome Studio V2011.1 (Methylation Module, version 1.9.1000).

### Karyotyping and immunohistochemistry of DIPG

Fresh tumor was disaggregated mechanically and enzymatically using collagenase V (Sigma-Aldrich, St. Louis, MO). The suspension cultures were incubated overnight or 24 h before harvest, and in-situ cultures were harvested after 3–12 days in culture. Karyotype was interpreted according to the International System for Human Cytogenetic Nomenclature (ISCN 2013). Immunohistochemical studies were performed as previously published using the Discovery XT processor (Ventana Medical Systems) [40, 41]. In brief, immunostaining was performed using the rabbit polyclonal anti-H3K27me3 (07–449, Millipore, Billercia, MA; 1 μg/mL) or rabbit polyclonal anti-H3.3 K27 M (ABE419, Millipore, Billercia, MA; 0.5 μg/mL) antibodies. Streptavidin- HRP and DAB detection kit (Ventana Medical Systems) were used according to the manufacturer instructions.

### Exome and transcriptome profiling of DIPG

For cases 1 and 3, frozen DIPG tumor and normal brain tissue from autopsy were submitted for whole exome (paired tumor and germline DNA) and transcriptome (tumor RNA) sequencing. Clinically-integrated sequencing was performed according to previously published methodology [26]. Nucleic acid preparation, high-throughput sequencing, and computational analysis were performed by the Michigan Center for Translational Pathology sequencing laboratory using standard protocols in adherence to the Clinical Laboratory Improvement Amendments.

For case 6, only frozen DIPG tissue (no germline DNA sample) was submitted for whole exome sequencing (WES). This sample was processed through the GATK 3.6 variant analysis pipeline as a germline sample. After variants were called, variant annotation using SnpEff was completed to assign mutation information. This annotated VCF file was filtered using bcftools on a known set of specific genes/histones of interest to examine the mutational landscape. These gene specific variants then were examined further based on mutation effect (high, moderate) as to elucidate the presence of any non-synonymous variants, processed to remove common variants with ≥5% allele frequencies in 1000 Genomes Project (2015) [18], NHLBI Exome Sequencing Project 6500 (ESP6500) [16], Exome Aggregation Consortium dataset (http://exac.broadinstitute.org), and Genome Aggregation Database (http://gnomad.broadinstitute.org), and removed of non-recurrent somatic variants using annotations in the COSMIC v70 database (http://cancer.sanger.ac.uk/cosmic).

### Mutational signature

The somatic mutations in each of the DIPG samples from cases 1 and 3 processed through MiOncoseq sequencing platform [26] were categorized into one of the 96 possible categories: 6 classes of base substitution (C > A, C > G, C > T, T > A, T > C and T > G) × 16 combinations of bases immediately 5′ and 3′ to each mutation base (context information), and the frequency of each mutation category per sample was computed [2, 3]. The previously defined 30 mutational signatures were downloaded from COSMIC (http://cancer.sanger.ac.uk/cosmic/signatures). Assuming the mutational distribution of a single sample is a linear combination of the known 30 signatures, an iterative method was used that was implemented in a R package deconstructSigs [35] to decompose the mutational signatures (a 96 × 30 matrix) for the observed mutational distribution of each DIPG sample (a 96 × 1 vector). The contributions of each known mutational signature in cases 1 and 3, the radiation-associated DIPG samples, were compared to all other primary cases, which were taken from both diagnosis and autopsy (*n* = 9).

### Statistical analysis

To estimate cumulative incidence of DIPG in survivors of pediatric medulloblastoma, the number of observed cases of DIPG during each observation period was divided by the total number of patients who underwent treatment of medulloblastoma [37]. For estimates of cumulative incidence from single institutions, data was obtained for the time period of January 2000 to December 2015. Survival data were extracted from the IDIPGR. Survival functions were estimated using Kaplan-Meier methods (GraphPad Prism version 7.00). For survival by histone status in primary DIPGs, analysis was limited to the subset of tumors for which the OS and sequencing information were available. Cox proportional hazards regression model was used to investigate the association between radiation exposure or histone status and survival after controlling for potential prognostic factors including age and sex (PROC PHREG in SAS 9.4). Mann Whitney test was performed using GraphPad Prism (version 7.00) to compare mutation and fusion frequency in DIPG in radiated and non-radiated setting.

## Results

Twelve patients who developed DIPG after radiation treatment for primary pediatric medulloblastoma were identified. Six of these cases were acquired from the IDIPGR, and six were extracted from literature review, reported primarily in results from medulloblastoma cooperative group trials: COG A9961 (*n* = 2), HIT’91 (*n* = 1), HIT-SIOP-PNET4 (*n* = 1), and CCG 9892 (*n* = 1) [12, 30, 31, 36, 42, 46]. Within the limits of incomplete follow-up timing and records, the estimated cumulative incidence of DIPG after medulloblastoma ranged from 0.3–3.9% among the involved institutions and reported studies (Table 1). The cumulative incidence of radiation-associated DIPG survivors of the reported trials ranged from 0.3–1.5% with median follow-up of 4.7–10 years, while the estimated cumulative incidences at single institutions ranged from 0.7–3.9%.

**Table 1:** Observed cumulative incidence of radiation-associated malignancies in survivors of pediatric medulloblastoma

Source	Population	Size of cohort	Number of secondary malignant neoplasms	Number of gliomas	Number of DIPG (cumulative incidence %)	Median follow-up (years)
COG A9961 (Packer et al. 2013) [31]	December 1996–2000, age 3–21 years, average-risk only	397	15	7	2 (0.5)	9.7
HIT’91 (Von Hoff et al. 2009) [42]	August 1991–December 1997, age 3–18 years	280	12	4	1 (0.4)	10
HIT-SIOP-PNET4 (Sabel et al. 2016) [36]	2001–2006, age 4–21 years, average risk only	338	3	2	1 (0.3)	7.8
CCG 9892 (Packer et al. 1999) [30]	January 1990–December 1994, age 3–10 years, average risk only	65	1	1	1 (1.5)	4.7
Single institution (Michigan Medicine)	Pediatric patients diagnosed with medulloblastoma and treated between 2000 and 2015	77	*Not reported*	*Not reported*	3 (3.9)	*Not reported*
Single institution (Seattle Children’s Hospital)	Pediatric patients diagnosed with medulloblastoma and treated between 2000 and 2015	91	*Not reported*	*Not reported*	1 (1.0)	*Not reported*
Single institution (Hospital for Sick Children)	Pediatric patients diagnosed with medulloblastoma and treated between 2000 and 2015	140	*Not reported*	*Not reported*	1 (0.7)	*Not reported*
Single institution (Princess Margaret Hospital for Children)	Pediatric patients diagnosed with medulloblastoma and treated between 2000 and 2015	41	*Not reported*	*Not reported*	1 (2.4)	*Not reported*

### Primary Medulloblastoma

Patient characteristics and treatments are described in Table 2. Of patients with known sex and age information (*n* = 7), six were male, and ages at diagnosis of primary medulloblastoma ranged from 2 to 9 years. For risk stratification of medulloblastoma based on clinical criteria, seven cases were average-risk, three were high-risk, and two were unreported. All cases with known histology (*n* = 6) showed classic histology (cases 1–6) with subgroup classification into either Group 3 (cases 3 and 4) or Group 4 (cases 1, 5, and 6). Cytogenetics was not performed on case 2, which was classified as Group 3/4 by immunohistochemistry (IHC).

**Table 2:** Characteristics of survivors of pediatric medulloblastoma who developed radiation-associated DIPG

	Primary Medulloblastoma				Radiation-associated DIPG				
Case number - location	Age at diagnosis (years)	Gender	Risk stratification	Histology, subgroup	Time in remission (years)	Age at diagnosis (years)	Treatment	Histology (sequencing)	Outcome (months after DIPG diagnosis)
1 - IDIPGR (Michigan Medicine)	8	F	Average	Classic, Group 4 (isochromosome 17q)	12	21	54 Gy focal radiation treatment, panobinostat	High-grade glioma (*TP53* loss, *PTEN* loss, *NRAS* mutation)	Died of disease (17)
2 - IDIPGR (Michigan Medicine)	9	M	Average	Classic, Group 3/4	7	16	Patient declined	Biopsy deferred	Died of disease (4)
3 - IDIPGR (Michigan Medicine)	4	M	High	Classic, Group 3	4	9	35 Gy focal radiation treatment, everolimus	High-grade glioma (*PIK3CA* and *EZH2* mutations)	Living (5)
4 - IDIPGR (Hospital for Sick Children)	2	M	High	Classic, Group 3	2	7	Etoposide, temozolomide, mechlorethamine, cyclophosphamide	*Not reported*	Died of disease (5)
5 - IDIPGR (Seattle Children’s Hospital)	6	M	Average	Classic, Group 4	10	17	Temozolomide	Not biopsied; diagnosis made by imaging	Died of disease (10)
6 - IDIPGR (Princess Margaret Hospital for Children)	4	M	High	Classic, Group 4	11	15	Focal radiation treatment, vorinostat	High-grade glioma *(PIK3CA* mutation)	Died of disease (8)
7- Packer et al. 2013 [31] (COG A9961)	*3–21*	*Not reported*	Average	*Not reported*	6.5	*Not reported*	*Not reported*	Pilocytic astrocytoma^a^	Died of disease (10)
8 - Packer et al. 2013 [31] (COG A9961)	*3–21*	*Not reported*	Average	*Not reported*	9	*Not reported*	*Not reported*	Biopsy deferred	*Not reported*
9 - Von Hoff et al. 2009 [42] (HIT’91)	*3–18*	*Not reported*	*Not reported*	*Not reported*	*Not reported*	*Not reported*	*Not reported*	*Not reported*	*Not reported*
10 - Sabel et al. 2016 [36] (HIT-SIOP-PNET4)	*4–21*	*Not reported*	Average	*Not reported*	5.1	*Not reported*	*Not reported*	Anaplastic astrocytoma	Died of disease (Not reported)
11 - Packer et al. 1999 [30] (CCG 9892)	*3–10*	*Not reported*	Average	*Not reported*	4.8	*Not reported*	*Not reported*	Glioma, grade unspecified	Died of disease (Not reported)
12 - You et al. 2013 [46] (Yonsei Hospital)	8	M	*Not reported*	*Not reported*	7.8	*Not reported*	Temozolomide	Anaplastic astrocytoma	Died of disease (6)

^a^Pathology was not reviewed centrally by trial

Treatment details are described in Additional file 1: Table S1. All cases underwent surgical resection. Seven cases achieved gross total resection and three cases had partial resections; surgical details were not reported for two cases. All patients received CSI (dose range 18.0–36.0 Gy) with a posterior fossa boost (dose range 19.8–36.0 Gy) for total posterior fossa exposure 53.4–60.0 Gy. Ten cases received chemotherapy, which included a variety of established regimens including cytotoxic and/or targeted therapies, one case declined chemotherapy, and one case lacked chemotherapy details.

### Radiation-associated DIPG

**Fig. 1:**
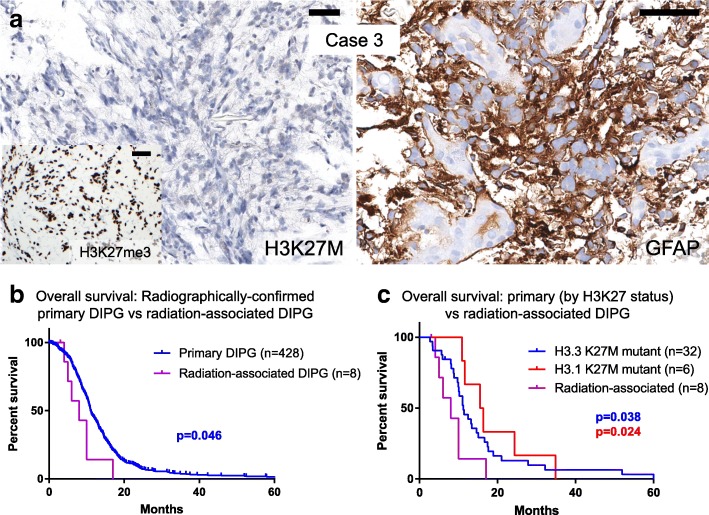
Radiation-associated DIPGs define a distinct molecular subtype with poor prognosis. **a** Immunohistochemistry performed for case 3 showed wild-type status for histone H3 (H3K27M) with retention of H3K27me3, as well as diffuse GFAP expression, which was negative in primary medulloblastoma (not shown). Positive and negative controls are shown in Additional file 1: Figure S1. Insufficient tissue was available for such analysis in cases 1 and 6; however, H3 wild-type status was demonstrated by tumor sequencing in these cases. All scale bars are 50 μm. **b** OS data for primary DIPG in the IDIPGR (*n* = 428) was compared via Kaplan-Meier analysis to OS of radiation-associated DIPG cohort (*n* = 8). OS was significantly less for the radiation-associated DIPG group (*p* = 0.046). **c** OS data for primary DIPG patients with both genomic and OS information available (*n* = 38), as categorized by histone mutational status and compared via Kaplan-Meier analysis to OS of radiation-associated DIPG cohort (*n* = 8). The radiation-associated DIPG cohort showed the shortest OS in comparison to the two subgroups of primary DIPG with significantly shorter survival compared to H3.3 K27 M mutant (*p* = 0.038) and H3.1 K27 M mutant (*p* = 0.024) primary DIPGs

Diagnostic and outcome information on DIPG for all cases are described in Table 2. Median time to diagnosis of DIPG after completion of primary medulloblastoma therapy was 7 years (range 2–11 years). DIPG histologic diagnoses were reported for 7 patients and included HGG (*n* = 3), glioma, grade-unspecified (*n* = 1), anaplastic astrocytoma (*n* = 2), and pilocytic astrocytoma (*n* = 1; case was not reviewed centrally by trial). For cases with tissue (cases 1, 3 and 6), IHC revealed the tumors to be negative for H3K27 M staining and GFAP positive (Fig. 1a, positive and negative control tumor staining in Additional file 1: Figure S1). Additional staining in case 3 revealed retention of H3K27me3 (Fig. 1a), consistent with previous analyses of H3 wild-type DIPG, as tri-methyl is lost in H3K27M-mutant glioma [6]. Treatment for DIPG varied widely. Three patients received focal EBRT, two of whom received oral treatment with histone deacetylase inhibitors (panobinostat, vorinostat) and one of whom received everolimus. Three additional patients received chemotherapeutic agents, with temozolomide being the most common choice. One patient declined treatment, and treatment details were not available for five patients.

For cases with complete outcome data (*n* = 8), seven patients died at a median of 8 months after DIPG diagnosis (range 4–17 months), and one patient remains living 5 months after DIPG diagnosis. OS was shorter for radiation-associated DIPG as compared to radiographically-confirmed primary DIPG cases from IDIPGR (*n* = 428, *p* = 0.046; Fig. 1b). On multivariate analysis, radiation exposure (hazard ratio 2.87; *p* = 0.014) and age (hazard ratio 1.00; *p* = 0.019) were associated significantly with overall survival (Additional file 1: Table S2). Further, the radiation-associated DIPG cohort showed the shortest OS compared to two subgroups of primary DIPG with sequencing information (*n* = 38), separated by H3 status (vs. H3.3 K27 M mutant, *p* = 0.038; vs. H3.1 K27 M mutant, *p* = 0.024; Fig. 1c). On multivariate analysis, radiation exposure (hazard ratio 4.51; *p* = 0.005) and sex (hazard ratio 2.51; *p* = 0.016) were associated significantly with overall survival (Additional file 1: Table S2).

**Fig. 2:**
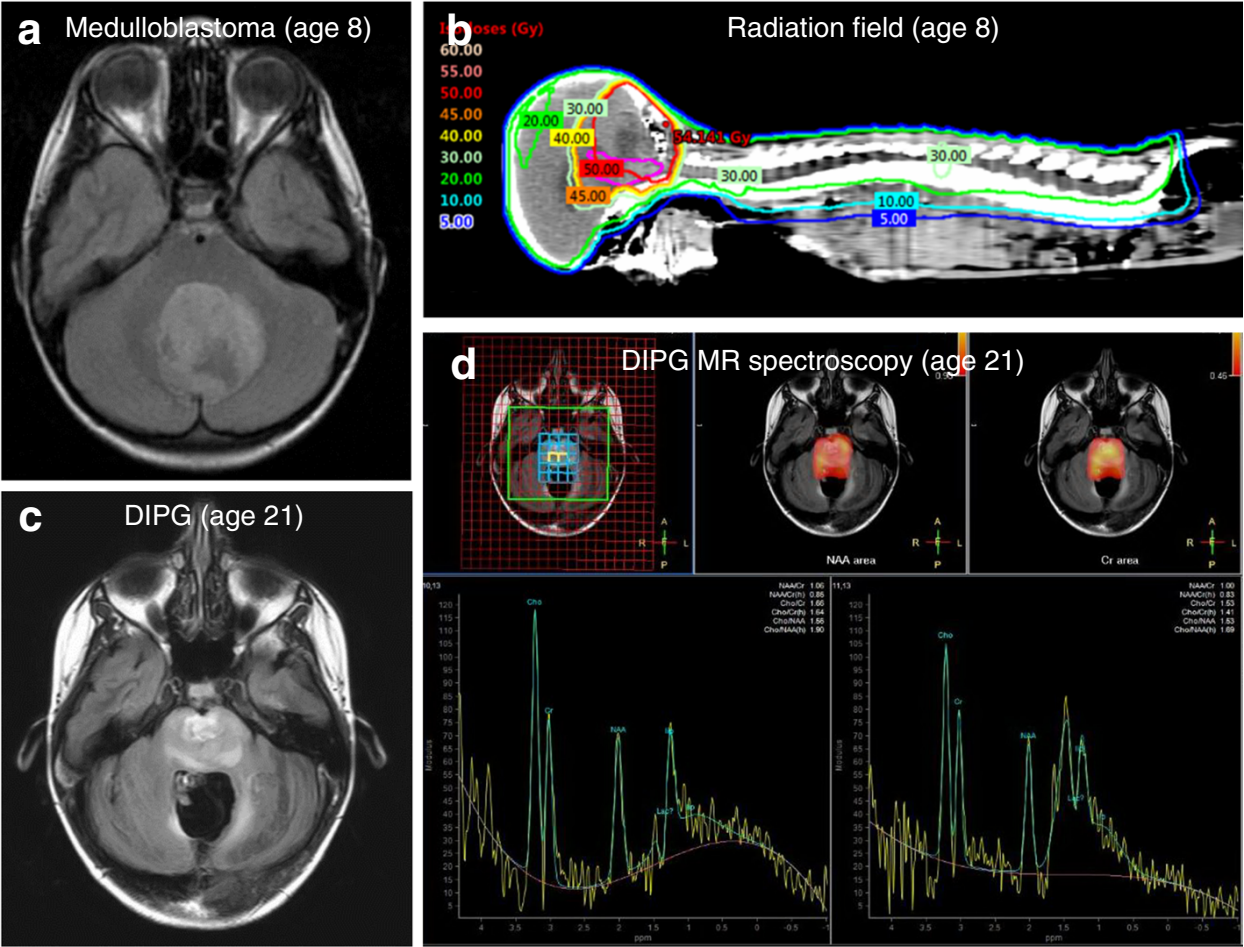
Diagnosis and treatment with standard therapy of case 1, which included significant radiation to brainstem. **a** MR axial T2 FLAIR image of primary medulloblastoma diagnosed at age 8. **b** Radiation dose distribution showing craniospinal irradiation prescribed to 23.4 Gy and posterior fossa boost prescribed to 32.4 Gy. The brainstem is contoured in purple and received a mean dose of 50.1 Gy. **c** MR axial T2 FLAIR image of DIPG diagnosed at age 21, 13 years after treatment for primary medulloblastoma and in the area of the previously irradiated field. **d** MR spectroscopy with an elevated Chol/Cr ratio (1.66) that is consistent with malignancy (DIPG)

**Fig. 3:**
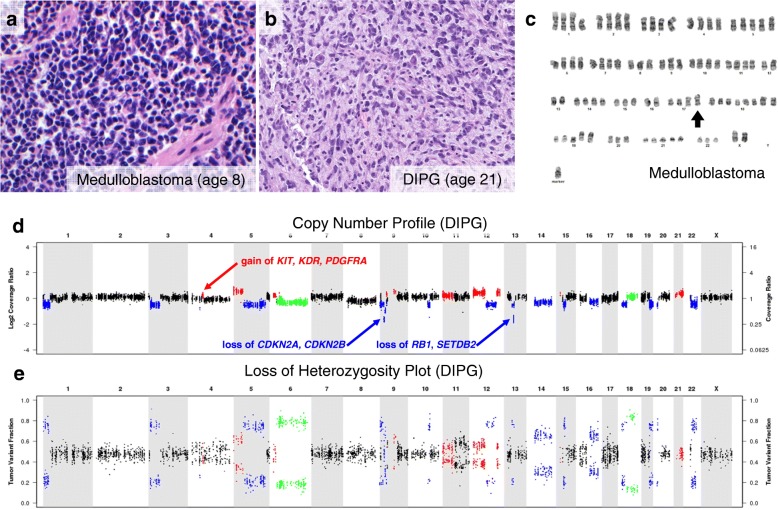
Histology and molecular results distinguish primary medulloblastoma from radiation-associated DIPG. **a** Resected medulloblastoma from case 1 showing characteristic classic-type features including sheets of cells with primitive hyperchromatic nuclei and scant cytoplasm. **b** DIPG at autopsy showing an infiltrating glial tumor with small angulated nuclei and abundant amphophilic cytoplasm. **c** Karyotype analysis of medulloblastoma shows near-tetraploid clone with arrow indicating i(17q), most consistent with Group 4. **d** Copy number analysis of DIPG shows focal and structural changes distinct from primary tumor, including focal homozygous loss of *RB1, SETDB2, CDKN2A* and *CDKN2B*, focal 1 copy gain of *KIT*, *KDR* and *PDGFRA*, and activation mutations in *NRAS* and *TP53*. **e** Loss of heterozygosity plot showing regions on chromosomes 6 and 18 with copy-neutral loss of heterozygosity events

For illustration, case 1 presented with standard-risk medulloblastoma at age 8 (Fig. 2a), and underwent gross total resection (Fig. 3a), followed by 23.4 Gy CSI with a 32.4 Gy boost to primary tumor site (Fig. 2b) with concurrent vincristine. At 21 years of age (12 years after completion of therapy), she presented to her primary care physician with a one-month history of difficulty swallowing and clumsiness of her right hand. MRI brain revealed a new infiltrative mass with diffuse pontine T2 hyperintensity, consistent radiographically with a DIPG (Fig. 2c). MR spectroscopy revealed markedly elevated choline to creatinine peak with depressed NAA peak, consistent with malignancy (Fig. 2d). She underwent re-irradiation and ultimately died of disease 17 months after diagnosis. Histopathology at autopsy revealed a diffusively infiltrating glioma (Fig. 3b). Karyotyping of her initial medulloblastoma previously had revealed isochromosome for the long arm of chromosome 17, a hallmark feature of Group 4 medulloblastoma (Fig. 3c). In contrast, copy number analysis of her DIPG revealed copy number changes frequently seen in gliomas including homozygous loss of *RB1*, *SETDB2*, *CDKN2A* and *CDKN2B* with no abnormalities in chromosome 17, distinguishing it from the primary medulloblastoma (Fig. 3d-e). Similar imaging findings, radiation fields and MR spectroscopy images were obtained in cases 2 and 3 (Additional file 1: Figures S2 and S3).

### DIPG sequencing

**Fig. 4:**
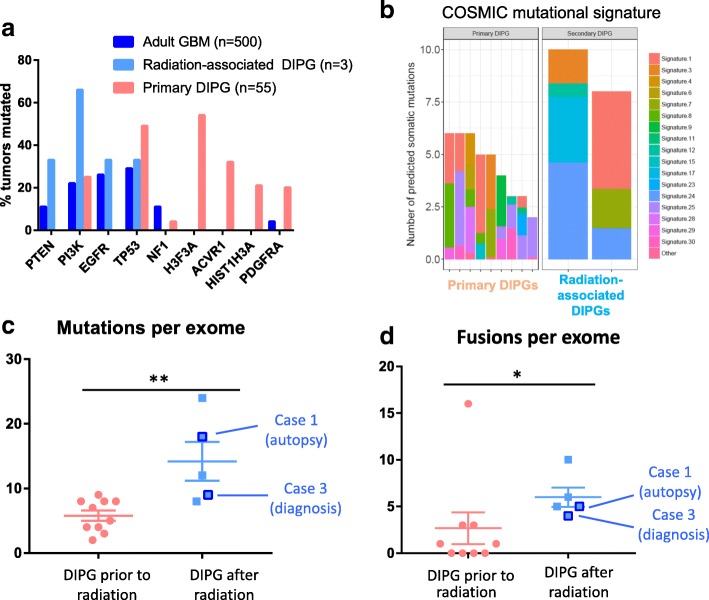
Radiation-associated DIPGs are molecularly distinct from primary DIPGs. **a** Plot of recurrent mutations in previously published datasets (adult GBM [*n* = 500] [7]; primary DIPG [*n* = 55] [44]) demonstrates that the distribution of driving mutations in radiation-associated DIPG is more similar to recurrent alterations in adult GBM than primary DIPG. **b** Contributions of established COSMIC mutational signatures were determined for radiation-associated DIPG samples as compared to all other primary cases sequenced through same sequencing platform (MI-ONCOSEQ). **c-d** Cases with previous radiation in this cohort (including case 1 and 3 and primary DIPG at autopsy) show higher mutations and fusions per exome (*p* = 0.0043 and *p* = 0.0135, respectively using Mann Whitney test)

For cases 1, 3, and 6, DIPG frozen tissue from autopsy (cases 1 and 6) and diagnosis (case 3) were sequenced and mutations were analyzed (Additional file 1: Table S3). All three tumors were wild-type for *H3F3A* and *HIST1H3B*. Mutational signature analysis of DIPGs showed mutations consistent with radiation-induced DNA damage (e.g., insertional event in *TP53*), as well as mutations in other oncogenic drivers (e.g., *PTEN*, *EGFR*, and *NRAS*), suggestive of a distinct mutational process as compared with primary DIPGs. Mutations identified in radiation-associated DIPGs had molecular overlap with recurrent drivers of adult GBM, using previously published datasets of adult GBM and primary DIPG (Fig. 4a) [7, 44]. Within a cohort sequenced using the same sequencing methodology (UM MI-ONCOSEQ) (*n* = 11), COSMIC mutational signature analysis demonstrated that radiation-associated DIPGs had the highest predicted somatic mutation counts and were more likely to harbor Signature 24 than primary DIPGs, which has not previously been connected to previous malignancy or radiation exposure (Fig. 4b) [3]. Cases with previous radiation in the MI-ONCOSEQ cohort (including cases 1 and 3 form this series as well as primary DIPG cases at autopsy) harbored higher mutations (Fig. 4c; *p* = 0.0043) and fusions per exome (Fig. 4d; *p* = 0.0135), although overall mutational burden remained lower than what might be clinically significant in comparison to cancers with known mismatch repair deficiency [22]. Cases 1 and 3 underwent germline sequencing as well, which revealed no evidence of cancer predisposition syndromes.

## Discussion

With cumulative incidences ranging from 4.2–12%, long-term survivors of medulloblastoma show an increased risk of central nervous system SMNs, particularly gliomas [15, 19, 29, 31, 39, 42]. The risk of glioma has been shown to increase linearly with radiation dose, with reported excess relative risk of 0.079–0.33 per Gy [4, 38]. Prior studies have commented on radiation-associated DIPG following individual cases of pediatric central nervous system cancers [1, 8, 9, 17], although no studies have commented specifically on the incidence or molecular characteristics of radiation-associated DIPGs following treatment for pediatric medulloblastoma. In standard medulloblastoma therapy, the brainstem receives high doses of EBRT due to its anatomic proximity to the posterior fossa boost. In this study, the estimated cumulative incidence of DIPG in children diagnosed with medulloblastoma and treated with EBRT ranged from 0.3–3.9%. The cumulative incidence reported in this study may have been impacted by incomplete or brief follow-up and may be underestimated as the cohort continues to age. While DIPG was diagnosed at a median of 7 years after completion of treatment for medulloblastoma, median follow-up was only 10 years or less for the cited studies. In large studies of pediatric survivors, median time to diagnosis of radiation-associated gliomas ranged from 6.6–17.4 years [9, 15, 28, 32, 38, 39]. Furthermore, in patients with treated primary medulloblastoma, posterior fossa tumors often are labeled as recurrent medulloblastomas based solely on radiographic evidence. It may be that some tumors that are not biopsied and assumed to be recurrent medulloblastomas may in fact be DIPGs.

Increasing attention has been given to the impact of radiation field and modality on efficacy and risk of SMNs. For medulloblastoma treatment, many centers now are shifting away from a posterior fossa boost and toward a primary site boost only [24, 43]. Preliminary results from a recently closed phase III COG trial (ACNS0331) of involved field radiotherapy with chemotherapy in average-risk medulloblastoma found no difference in 5-year event free survival or OS when boost volume was limited to the primary site vs. entire posterior fossa [25]. In patients treated with primary site boost only, proton radiotherapy may decrease brainstem radiation exposure even further relative to photon therapy [11, 34]. In a multi-institutional cohort study and phase II single center trial, there were no significant differences in recurrence-free survival or OS between patients treated with photon vs. proton radiotherapy, and in the phase II trial, no radiation-associated malignancies were reported within a median follow-up time of 7 years [45]. While longer follow-up is required to evaluate definitively its impact, smaller boost fields and proton radiotherapy show promise for reducing the risk of SMNs without sacrificing efficacy of treatment.

Radiation-associated gliomas are molecularly distinct from their primary counterparts. A previous report of non-brainstem radiation-associated pediatric GBM showed overexpression of a number of genes involved in tumorigenesis as compared to primary pediatric GBM [13]. Additionally, prior studies suggest that tumors can be differentiated based on these molecular signatures and that radiation-associated tumors may exhibit distinct patterns [3, 5]. It has been observed that radiation-associated tumors exhibit a significantly higher total number of mutations, as well as balanced inversions, with both small deletions and inversions generating driver mutations [5].

In this study, tumor exome sequencing of three radiation-associated DIPGs demonstrated tumors to be H3-wildtype. This finding is significant in the context of a recent large cohort of sequenced primary DIPGs, in which only 16.8% were found to be H3-wildtype [23]. Notably, sequencing confirmed that the tumors were indeed distinct from their primary malignancies and not local recurrences. Moreover, patients did not harbor germline mutations in known cancer predisposition genes. Although alterations in two of the most frequently mutated genes in primary DIPG (*H3F3A* and *ACVR1*) were not detected, the tumor mutations in the sequenced cases are established tumor drivers in adult GBM (e.g. *PTEN*, *NRAS*, and *EGFR*). Interestingly, case 3 was found to have an *EZH2* mutation in the radiation-associated DIPG, which has not been identified as a recurrent driver in DIPG, but has been established as a potential therapeutic target in pre-clinical DIPG models [27]. Mutational signature analysis of radiation-associated DIPGs showed mutations consistent with radiation-induced DNA damage (e.g., insertional event in *TP53*), at a rate similar to cases observed in a recent report of radiation-associated GBMs, in which two out of five sequenced GBMs had mutations in *TP53* [33]. Unlike radiation-associated GBMs, however, *PDGFRA* played a smaller role in the cohort of radiation-associated DIPG. The *PDGFRA* amplification in case 1 (Fig. 1d) was the isolated alteration in the three tumors. While non-silent mutations in *PDGFRA* were identified in all five radiation-associated GBMs, none of radiation-associated DIPGs from this study had mutations in *PDGFRA*.

Taken together, these results are suggestive of distinct mutational processes compared with primary DIPGs: primary DIPGs originate within a particular early developmental timespan that is amenable to transformation with *H3F3A* and *ACVR1* mutation [23], whereas radiation-associated DIPGs appear to arise as a result of radiation-induced DNA damage in established oncogenic drivers of primary adult GBM. Future sequencing of additional cases may elucidate patterns of distinct biology in radiation-associated DIPG, which may have implications in terms of clinical management. These data suggest that patients with radiation-associated DIPG may benefit from future therapies targeted to the molecular features of adult GBM rather than primary DIPG.

Significantly, the radiation-associated DIPG cohort demonstrated a shorter OS relative to patients with primary DIPG. The three molecularly sequenced cases of radiation-associated DIPG cohort additionally are distinguished as H3 wild-type designation, considered a positive prognostic variable in primary DIPG relative to H3.3 mutant DIPG [10, 20]. Prior studies report a similar decreased survival from radiation-induced HGG as compared to primary HGG [13, 21]. In conjunction with the molecular findings, these data suggest that radiation-associated DIPGs form a distinct molecular subgroup that has negative implications on survival. The findings in this work demonstrate the importance of tumor biopsy or resection at appearance of a second cancer. Most cases had no or limited histological and molecular diagnostic information, which was not clinically relevant until recently. Now, this information is critical for prognostic information, current clinical management, and potential future therapies [33].

## Conclusions

In conclusion, patients treated for pediatric medulloblastoma are at increased risk for development of radiation-associated DIPG, which may represent a distinct molecular subtype with a worse prognosis relative to other DIPGs. This risk highlights the importance of radiation volume and modality in the treatment of children with medulloblastoma and provides a compelling argument for efforts to reduce exposure of the brainstem. Additionally, the presented molecular data suggest that patients with radiation-associated DIPG may benefit from future therapies targeted to the molecular features of adult GBM rather than primary DIPG.

## Additional file

Additional file 1: **Table S1.** Treatment details for primary medulloblastoma. **Table S2.** Multivariate analysis of overall survival for primary and radiation-associated DIPGs. **Table S3.** Sequencing of radiation-associated DIPGs. **Figure S1.** Immunohistochemical staining for H3K27 M of positive and negative control pediatric high-grade gliomas. **Figure S2.** Diagnosis and management of case 2, which included non-standard treatment for medulloblastoma. **Figure S3.** Diagnosis and management of case 3, which included standard therapy for medulloblastoma. (DOCX 5366 kb)
