# Supplementary material for: Medulloblastoma therapy generates risk of a poorly-prognostic H3 wild-type subgroup of diffuse intrinsic pontine glioma: a report from the International DIPG Registry
Source: Acta Neuropathol Commun. 2018 Jul 26;6:67. doi: 10.1186/s40478-018-0570-9 (PMC6062866; doi:10.1186/s40478-018-0570-9)
Supplement: Supplementary file 1 — Table S1. Treatment details for primary medulloblastoma. Table S2. Multivariate analysis of overall survival for primary and radiation-associated DIPGs. Table S3. Sequencing of radiation-associated DIPGs. Figure S1. Immunohistochemical staining for H3K27 M of positive and negative control pediatric high-grade gliomas. Figure S2. Diagnosis and management of case 2, which included non-standard treatment for medulloblastoma. Figure S3. Diagnosis and management of case 3, which included standard therapy for medulloblastoma. (DOCX 5366 kb) [file 40478_2018_570_MOESM1_ESM.docx]

**Electronic Supplementary Material**

Contents:

Table S1

Table S2

Table S3

Fig. S1

Fig. S2

**Table S1**

**Treatment details for primary medulloblastoma**

| **Case number - location** | **Extent of resection** | **Craniospinal irradiation dose (Gy) (dose per fraction [Gy])** | **Posterior fossa boost (Gy) (dose per fraction [Gy])** | **Total posterior fossa exposure (Gy)** | **Mean brainstem exposure (Gy) (Maximum point dose [Gy])** | **Chemotherapy regimen** |
| --- | --- | --- | --- | --- | --- | --- |
| 1 - IDIPGR (Michigan Medicine) | Gross total resection | 23.4 (1.8) | 32.4 (1.8) | 55.8 | 50.1 (51.8) | COG A9961 regimen A: lomustine, vincristine, cisplatin |
| 2 - IDIPGR (Michigan Medicine) | Gross total resection | 30.6^a^ | 25.5^a^ | 56.1^a^ | 54.0 (55.5)^a^ | Patient declined |
| 3 - IDIPGR (Michigan Medicine) | Gross total resection | 36.0 (1.8) | 19.8 (1.8) | 55.8 | 56.4 (57.8) | ACNS 0332 arm C: concurrent weekly vincristine; adjuvant 6 cycles of cisplatin, cyclophosphamide, and vincristine with isotretinoin during maintenance and six months afterwards |
| 4 - IDIPGR (Hospital for Sick Children) | > 1.5 cm^3^ residual | 18.0 | 36.0 | 54.0 | 47.1 (54.9) | Initial: "Baby POG" (vincristine, cyclophosphamide, etoposide, cisplatin) and carboplatin, thiotepa, etoposide. Relapse: MOPP (mustargen, oncovin, procarbazine, prednisone), vincristine, lomustine. |
| 5 - IDIPGR (Seattle Children's Hospital) | Gross total resection | 23.5 | 30.6 | 54.1 | *Not reported* | CCG 9892: carboplatin, cisplatin, lomustine |
| 6 - IDIPGR (Princess Margaret Hospital for Children) | Partial resection | 36.0 | 19.8 | 55.8 | *Not reported* | COG 99701: concurrent daily carboplatin and weekly vincristine throughout 6 weeks of radiotherapy. Maintenance regimen B: cyclophosphamide, vincristine, cisplatin |
| 7- Packer et al. 2013 (COG A9961) | Gross total resection | 23.4 (1.8) | 32.4 (1.8) | 55.8 | *Not reported* | COG A9961 regimen B: cyclophosphamide, cisplatin, vincristine |
| 8 - Packer et al. 2013 (COG A9961) | Gross total resection | 23.4 (1.8) | 32.4 (1.8) | 55.8 | *Not reported* | COG A9961 |
| 9 - Von Hoff et al. 2009 (HIT'91) | *Not reported* | 35.2 (1.6) | 20 (2.0) | 55.2 | *Not reported* | HIT '91: vincristine, cisplatin, lomustine vs. ifosfamide, etoposide, high dose methotrexate, cisplatin, cytarabine |
| 10 - Sabel et al. 2016 (HIT-SIOP-PNET4)^b^ | Gross total resection | 23.4 or 36.0 (1.8 or 1.0) | 30.0 or 24.0 | 53.4 or 60.0 | *Not reported* | Cisplatin, vincristine, lomustine |
| 11 - Packer et al. 1999 (CCG 9892) | Gross total resection | 23.4 (1.8) | 31.8 | 55.2 | *Not reported* | Cisplatin, vincristine, lomustine |
| 12 - You et al. 2013 (Yonsei Hospital) | *Not reported* | 36.0 | 19.8 | 55.8 | *Not reported* | *Not reported* |

^a^Radiation treatment compromised by prolonged treatment interruption. Patient completed only 30.6 Gy of 36 Gy recommended craniospinal irradiation. ^b^Treatment arm was not specified in HIT-SIOP-PNET4 trial; included is the description of treatment arms for standard and hyperfractionated courses.

**Table S2**

**Multivariate analysis of overall survival for primary and radiation-associated DIPGs**

| **Parameter** | **Hazard ratio** | **95% hazard ratio confidence limits** | | **p** |
| --- | --- | --- | --- | --- |
|  |  |  | |  |
| *Full cohort* |  |  | |  |
| Radiation-associated | 2.87 | 1.24 | 6.65 | 0.014 |
| Age | 1.00 | 1.00 | 1.00 | 0.019 |
| Sex | 1.16 | 0.96 | 1.41 | 0.128 |
|  |  |  |  |  |
| *Cases with molecular data* |  |  |  |  |
| Radiation-associated | 4.51 | 1.59 | 12.78 | 0.005 |
| Age | 1.00 | 0.99 | 1.00 | 0.462 |
| Sex | 2.51 | 1.18 | 5.33 | 0.016 |
| H3.3 K27M mutant | 0.91 | 0.48 | 1.74 | 0.784 |
| Age | 1.00 | 1.00 | 1.00 | 0.987 |
| Sex | 2.40 | 1.16 | 4.98 | 0.018 |
| H3.1 K27M mutant | 0.66 | 0.27 | 1.61 | 0.358 |
| Age | 1.00 | 1.00 | 1.00 | 0.793 |
| Sex | 2.30 | 1.10 | 4.79 | 0.026 |

**Table S3**

**Sequencing of radiation-associated DIPGs**

| **Case number** | **Gene** | **Protein Change** | **Effect** | **Allelic Fraction (%)** | **dbSNP** |
| --- | --- | --- | --- | --- | --- |
| 1 | *LZTR1* | p.G405V | Missense | 41 | *Not available* |
|  | *ELF2* | p.E221A | Missense | 39 | *Not available* |
|  | *PTEN* | p.R130G | Missense | 36 | rs121909224 |
|  | *EPHB3* | p.E63D | Missense | 31 | *Not available* |
|  | *NRAS* | p.Q61K | Missense | 28 | rs121913254 |
|  | *MTCP1* | p.R76S | Missense | 26 | *Not available* |
|  | *SOX2* | p.Y227fs | Frameshift | 22 | *Not available* |
|  | *LZTR1* | p.L57P | Missense | 11 | *Not available* |
|  | *TP53* | p.C176fs | Frameshift; stop gain | 11 | *Not available* |
|  | *MGA* | p.L752fs | Frameshift | 10 | *Not available* |
|  | *CSNK1E* | p.P245F | Missense | 9 | *Not available* |
|  | *BRAF* | p.G596R | Missense | 9 | rs121913361 |
|  | *KDM5B* | p.T1044I | Missense | 9 | *Not available* |
|  | *RYR2* | p.L4376del | In-frame deletion | 9 | *Not available* |
|  | *EIF1AX* | p.K7R | Missense | 8 | *Not available* |
|  | *NOTCH1* | p.I1308fs | Frameshift | 8 | *Not available* |
|  | *IL6ST* | p.E234fs | Frameshift | 6 | *Not available* |
|  | *SLC6A3* | p.A18fs | Frameshift | 6 | *Not available* |
| 3 | *PIK3CA* | p.G106V | Missense | 43 | rs1057519930 |
|  | *CYP2C19* | p.T130M | Missense | 40 | rs150152656 |
|  | *EZH2* | p.D681N | Missense | 40 | *Not available* |
|  | *PIK3CA* | p.H1065L | Missense | 20 | *Not available* |
|  | *PIK3CA* | p.R88Q | Missense | 16 | rs121913287 |
|  | *PRRX1* | p.R95L | Missense | 9 | *Not available* |
|  | *MYH11* | p.R771* | Stop gain | 6 | *Not available* |
|  | *TCF12* | p.M400fs | Frameshift | 4 | *Not available* |
|  | *PIK3CA* | p.N714Y | Missense | 3 | *Not available* |
| 6 | *EPHA8* | p.R884H | Missense | 85 | rs62618734 |
|  | *GRK4* | p.R26C | Missense | 57 | rs146194528 |
|  | *FLG* | p.R971H | Missense | 47 | rs141120727 |
|  | *FLG* | p.E2074Q | Missense | 45 | rs146104019 |
|  | *BAZ2A* | p.R1793W | Missense | 45 | *Not available* |
|  | *PIK3CA* | p.H1047L | Missense | 38 | rs121913279 |
|  | *EGFR* | p.R791R | Structural interaction variant | 38 | rs2229066 |
|  | *CRTC3* | p.R70Q | Missense | 35 | *Not available* |
|  | *PRSS3* | p.K186E | Missense | 16 | rs200731425 |
|  | *KMT2C* | p.Q755* | Stop gain | 14 | rs201234598 |

**Fig. S1**

Immunohistochemical staining for H3K27M of positive and negative control pediatric high-grade gliomas.

Immunohistochemical studies were performed using the rabbit polyclonal anti-H3.3 K27M (ABE419, Millipore, Billercia, MA; 0.5 μg/mL) on (a) a pediatric high-grade glioma confirmed to by H3 K27M, and (b) H3 wildtype by sequencing of the *H3F3A* (H3.3) and *HIST1H3B* (H3.1) genes, to serve as positive and negative controls, respectively.

**Fig. S2**

Diagnosis and management of case 2, which included non-standard treatment for medulloblastoma

**a** MR axial T1 image with contrast of primary medulloblastoma diagnosed at age 9. **b** Radiation dose distribution showing craniospinal irradiation to 30.6 Gy and posterior fossa boost to 25.5 Gy. Note that due to the family’s request, the patient’s treatment course was non-standard. Radiation treatment was compromised by prolonged treatment interruption, and the patient completed only 30.6 Gy of 36 Gy recommended craniospinal irradiation. The brainstem is contoured in purple and received a mean dose of 54.0 Gy. **c** MR axial T2 FLAIR image of DIPG diagnosed at age 16, seven years after completion of medulloblastoma treatment and in the area of the previously irradiated field. **d** MR spectroscopy with an elevated Chol/Cr ratio (3.07) that is consistent with malignancy (DIPG).

**Fig. S3**

Diagnosis and management of case 3, which included standard therapy for medulloblastoma

**a** MR axial T1 image with contrast of primary medulloblastoma diagnosed at age 4. **b** Radiation dose distribution showing craniospinal irradiation prescribed to 36.0 Gy and posterior fossa boost prescribed to 19.8 Gy. The brainstem is contoured in purple and received a mean dose of 56.4 Gy. **c** MR axial T2 FLAIR image of DIPG diagnosed at age 9, four years after completion of medulloblastoma treatment and in the area of the previously irradiated field. **d** MR spectroscopy with an elevated Chol/Cr ratio (2.06) that is consistent with malignancy (DIPG).
